# False-positive treponemal syphilis serology linked to EBV-related heterophile antibodies: Insights from a multi-platform diagnostic

**DOI:** 10.1016/j.idcr.2025.e02403

**Published:** 2025-10-15

**Authors:** Mengjie Luo, Yi Wu, Qiling Lin, Chunlei Zhang

**Affiliations:** Shenzhen Yantian District People’s Hospital, Shenzhen, Guangdong 518081, China

**Keywords:** Syphilis, Epstein–Barr virus, False-positive, Heterophile antibodies

## Abstract

**Background:**

Syphilis serological false positives associated with interference from heterophile antibodies induced by Epstein–Barr virus (EBV) remain rarely reported. This report aims to document a rare case of syphilis serology discordance ultimately attributed to EBV infection, imitating syphilis serology.

**Case Presentation:**

A 34-year-old woman presented with facial skin lesions and underwent syphilis screening. Initial testing revealed reactive *Treponema pallidum* (*T. pallidum*) antibody (TP-Ab), positive *T. pallidum* particle agglutination assay (TPPA), and negative toluidine red unheated serum test (TRUST). Subsequent testing, including chemiluminescent platforms, immunofluorescence (FTA-ABS), Western blot, and colloidal gold methods, was non-reactive. Comprehensive workup for autoimmune and endocrine disorders was unremarkable. Further investigation revealed elevated antiphospholipid antibodies and positive EBV serologies. EBV DNA was detected. After heterophilic antibody blocking, TPPA and TP-Ab returned to negative, confirming false-positive results.

**Conclusion:**

This case illustrates that heterophile antibody interference can simultaneously affect both particle agglutination and chemiluminescence-based treponemal assays, leading to false-positive results. It emphasizes the necessity of interpreting serological findings in conjunction with clinical history and, when appropriate, confirmatory molecular testing, in order to prevent misdiagnosis and unnecessary treatment.

## Introduction

Syphilis, a major cause of adverse pregnancy outcomes in low and middle-income countries, remains a significant public health concern, with 438,000 cases reported in China in 2016 alone [1], [2]. Therefore, reducing the global incidence of syphilis by 90 % between 2018 and 2030 has been highlighted by the World Health Organization as one of the four ambitious goals [3], [4]. Its diagnosis relies heavily on serological tests, which typically involve both non-treponemal tests (NTTs) and treponemal tests (TTs), with the latter, such as the *T. pallidum* IgG chemiluminescence immunoassay (CLIA) and the *T. pallidum* particle agglutination (TPPA), being commonly used [5]. Syphilis serological false positives may lead to misdiagnosis, unnecessary treatment, and patient anxiety, commonly associated with autoimmune conditions or aging [6]. Biological false-positive reactions derived from heterophile antibody interference are well-documented in tumor marker assays and NTTs but rarely reported in TTs. However, given that the TTs serve as confirmatory tests in traditional syphilis diagnostic algorithms, more reports focused on the occurrence of false-positive TTs are required to provide a reference for clinicians and laboratory specialists. We describe a case in which a young female patient exhibited discordant syphilis serologic results, which were eventually attributed to EBV-related immune cross-reactivity.

## Case Presentation

**Fig. 1:**
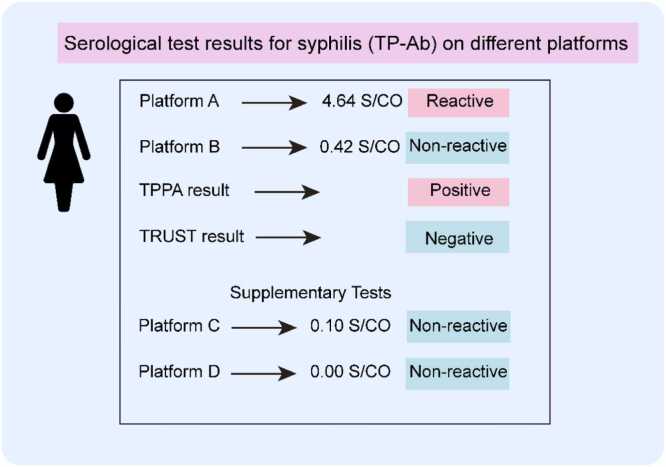
**Initial serological test results for syphilis on different platforms.** TP-Ab test result showed a reactive signal on Platform A, while showing a non-reactive result on Platform B, C, and D. TPPA result was positive, indicating potential treponemal exposure or false positivity. TRUST result was negative, suggesting no current active syphilis infection. The details of the test methods used for each platform are provided in the supplementary materials. Abbreviations: TP-Ab, *Treponema pallidum* Antibody; TPPA, *Treponema pallidum* particle agglutination; TRUST, toluidine red unheated serum test; S/CO, signal-to-cutoff ratio.

In early April 2025, a 34-year-old female presented to Hospital A with facial erythematous papules and pustules persisting for several months. She reported intermittent pain and ineffective prior dermatological treatments, including fruit acid application, with a 4-month history of facial papules and pustules unresponsive to topical therapy. She had no history of arthralgia, Raynaud's phenomenon, photosensitivity, alopecia, or other autoimmune disorders. She also denied any syphilis exposure or high-risk sexual behavior. Initial syphilis serology using chemiluminescent Platform A revealed reactive *T. pallidum* antibodies (TP-Ab) (Fig. 1), TPPA positive, and non-treponemal testing (TRUST) negative. Later the same day, she was referred to the gynecology department of a tertiary hospital for further evaluation, where repeat testing on Platform B showed a non-reactive TP-Ab (Fig. 1). To resolve this discrepancy, we repeated TPPA (still positive), TRUST (negative), and a colloidal gold assay (negative). Repeat tests, including serial dilutions and dual-review TRUST evaluations, were consistent with the initial findings. The details of the test methods used in this case are described in the supplementary materials. Her medical history was unremarkable: no known food or drug allergies, no history of viral hepatitis, diabetes, pregnancy, or autoimmune disease. Initial dermatological diagnoses included acne and folliculitis. Laboratory tests showed: hemoglobin 129 g/L, packed cell volume 38.8 percent, leukocytosis 5280/cu.mm, neutrophils 55.4 percent, lymphocytes 38 percent, eosinophils 0.8 percent. To further investigate platform-specific variability, samples were sent to two independent laboratories using Platforms C and D, both of which showed non-reactive TP-Ab. Further confirmatory testing, comprising indirect immunofluorescence, FTA-ABS immunoglobulin G (IgG) and immunoglobulin M (IgM), and Western blot IgG/IgM, was non-reactive across all assays.

A detailed serological follow-up over the subsequent months remained consistent with the initial findings. Additional investigations for autoimmune and endocrine abnormalities, including Antinuclear Antibody (ANA) profile, rheumatoid factor, and thyroid antibodies (see Supplementary Table S1), were all negative. During extended testing, we detected elevated antiphospholipid antibodies (aPL), including anticardiolipin IgM and β2-glycoprotein I IgM (Table 1). Serological testing for Epstein–Barr virus (EBV) revealed positivity for anti-VCA-IgA, and anti-EBNA-IgA, while retrospective testing of a stored serum sample collected in May demonstrated negative anti-VCA-IgM, and positive anti-VCA-IgG, anti-EA-IgM, and anti-EBNA-IgG (Table 1). In addition, quantitative EBV DNA testing of peripheral blood detected 9.53E + 02 copies/mL (reference range, <5.00E + 02 copies/mL) (Table 1). This serological profile is highly suggestive of a past EBV infection with possible reactivation, rather than a primary acute infection. A heterophilic antibody test was also positive. Notably, the blocking test results demonstrated that the positive reactions of TPPA and TP-Ab (Platform A) were indeed false positives, and that these false-positive results were caused by heterophile antibody interference, likely related to EBV infection. All serological tests were performed using automated chemiluminescent immunoassay platforms according to the manufacturers’ instructions. The patient's facial lesions, comprising red papules, pustules, and folliculitis, were consistent with a presentation of acneiform eruption. However, given the eventual diagnosis, it is plausible that the skin manifestations were related to EBV infection. EBV can induce skin lesions through mechanisms such as cutaneous vascular dilation and inflammatory cell infiltration, leading to various forms of rash, including maculopapular and urticarial eruptions. In southern China, an endemic region for nasopharyngeal carcinoma (NPC), these IgA-based antibodies are often used as supplementary markers of EBV activity. Therefore, VCA-IgA and EBNA-IgA were also tested in our case in addition to conventional EBV serological markers. The positive results in the patient raised the concern of a possible EBV-associated malignancy. However, the patient exhibited no clinical symptoms suggestive of NPC, and no imaging findings indicated a malignant process. Taken together, while the IgA serology indicated EBV exposure, the overall clinical context did not support an EBV-related tumor. Following a course of symptomatic management (including fruit acid treatment, doxycycline, and isotretinoin), the acneiform symptoms ameliorated, although residual erythematous papules and post-inflammatory hyperpigmentation persisted.

**Table 1:** Immunological evidence of serological interference: EBV-related antibodies and antiphospholipid antibodies detected in the patient.

Test Item	Result	Unit	Reference Interval	Methodology
Anti-EB-NA antibody -IgA	3.22	COI	< 0.9 Non-reactive ≥ 1.1 Reactive 0.9–1.1 Equivocal	Chemiluminescence Immunoassay
Anti-EB-NA antibody -IgG	> 600	U/mL	< 5 Non-reactive ≥ 20 Reactive 5–20 Equivocal	Chemiluminescence Immunoassay
Anti-EB-EA antibody -IgM	2.07	COI	< 0.9 Non-reactive ≥ 1.1 Reactive 0.9–1.1 Equivocal	Chemiluminescence Immunoassay
Anti-EB-VCA antibody -IgM	3.07	U/mL	< 20 Non-reactive ≥ 40 Reactive 20–40 Equivocal	Chemiluminescence Immunoassay
Anti-EB-VCA antibody -IgG	396.2	U/mL	< 20 Non-reactive ≥ 40 Reactive	Chemiluminescence Immunoassay
Anti-EB-VCA antibody -IgA	7.26	COI	< 0.9 Non-reactive ≥ 1.1 Reactive 0.9–1.1 Equivocal	Chemiluminescence Immunoassay
EBV DNA	9.53E + 02	copies/mL	< 5.00E + 02	Quantitative PCR
Anti-Beta−2-Glycoprotein 1 antibody-IgA	< 2.0	AU/mL	< 16 Non-reactive > 24 Reactive 16–24 Equivocal	Chemiluminescence Immunoassay
Anti-Beta−2-Glycoprotein 1 antibody-IgG	3.90	AU/mL	< 16 Non-reactive ≥ 24 Reactive 16–24 Equivocal	Chemiluminescence Immunoassay
Anti-Beta−2-Glycoprotein 1 antibody-IgM	117.80	AU/mL	< 16 Non-reactive ≥ 24 Reactive 16–24 Equivocal	Chemiluminescence Immunoassay
Anti-Cardiolipin antibody-IgG	3.10	GPLU/mL	< 8 Non-reactive ≥ 12 Reactive 8–12 Equivocal	Electrochemiluminescence Immunoassay
Anti-Cardiolipin antibody-IgM	24.00	MPLU/mL	< 8 Non-reactive ≥ 12 Reactive 8–12 Equivocal	Electrochemiluminescence Immunoassay
Anti-Cardiolipin antibody-IgA	< 2.5	APLU/mL	< 8 Non-reactive ≥ 12 Reactive 8–12 Equivocal	Chemiluminescence Immunoassay

Abbreviations: EBV, Epstein–Barr virus; EB-NA: Epstein–Barr nuclear antigen; EB-EA: Epstein–Barr early antigen; EB-VCA: Epstein–Barr viral capsid antigen; IgA/G/M: Immunoglobulin A/G/M; DNA: Deoxyribonucleic acid; PCR: Polymerase chain reaction; COI: Cut-off index; GPLU: IgG phospholipid unit; MPLU: IgM phospholipid unit; APLU: IgA phospholipid unit.

## Discussion

False-positive syphilis serology has long been recognized, especially in contexts such as autoimmune diseases, pregnancy, malignancies, and certain infections [7]. This case reported a rare but diagnostically challenging instance of false-positive treponemal serology, ultimately attributable to EBV-associated heterophilic antibody interference. The female patient was initially tested reactive for TP-Ab on chemiluminescence platform A, while TRUST remained consistently negative, and conflicting results were obtained when using a different TP-Ab platform. Such discordance between treponemal assays, especially between different chemiluminescence-based platforms, prompted further investigation into potential analytical and biological causes of serological interference. EBV is a known inducer of heterophilic antibodies, which can bind non-specifically to assay components and cause cross-reactivity in immunoassays [8], [9]. These heterophilic antibodies can mimic the binding characteristics of pathogen-specific antibodies, thereby generating false-positive results in antigen-antibody detection systems [10]. Previous reports have documented EBV-associated false positivity in treponemal tests. A recent case series described three patients with EBV-infectious mononucleosis (EBV-IM) who simultaneously exhibited false positives in both non-treponemal and treponemal assays [11]. Similarly, another case reported concurrent false-positive treponemal serology in patients with EBV-IM, complicating syphilis screening in primary care settings [12].

In this case, the serological profile showed positive VCA-IgA, VCA-IgG, EBNA-IgA, EBNA-IgG, and EA-IgM, with negative VCA-IgM, combined with an EBV DNA viral load of 9.53E + 02 copies/mL (above the normal threshold). This pattern rules out a primary EBV infection and indicates a past EBV infection with possible reactivation [13], [14]. Classical heterophile antibody production is most documented during primary EBV infection due to the activation of polyclonal B-cells. However, heterophile antibodies have been reported to persist at low levels for several months to approximately one year after acute infectious mononucleosis [8], [15]. The detectable EBV DNA and elevated viral load support biologic activity that could plausibly contribute to heterophile-mediated assay interference [9]. Concurrently, the patient also tested positive for anti-cardiolipin IgM and β2-glycoprotein I IgM. It is well-established that these aPL are a common cause of biological false positives in NTTs like RPR and TRUST [16]. Notably, aPL-mediated false positives in NTTs have been frequently associated with autoimmune conditions like systemic lupus erythematosus (SLE) and antiphospholipid syndrome (APS), as well as various infections [17], [18]. However, in our case, the TRUST was consistently negative, effectively ruling out aPL as a direct cause of the observed serological discordance. Instead, the presence of aPL, alongside the confirmed EBV infection, is more like a serological marker of the broader polyclonal B-cell activation [19]. This state of non-specific immune stimulation is the likely prerequisite for the production of heterophile antibodies. Importantly, after applying the heterophilic antibody blocking reagent, both TP-Ab and TPPA reactivity were non-reactive, further implicating non-specific serum factors in the generation of false-positive results. This observation aligns with prior reports that interference derived from heterophilic antibodies can be corrected by specific blocking reagents, confirming the analytical nature of the interference rather than true serological evidence of *T. pallidum* infection [20], [21].

In addition to EBV-associated heterophile antibody interference observed in the present case, TTs false positivity results have also been sporadically reported in other pathological states. Autoimmune diseases such as SLE and APS have been implicated, likely due to polyclonal B-cell activation and the presence of autoantibodies with cross-reactivity to treponemal antigens [7], [22]. Infectious diseases, including Lyme disease and HIV infection, have also been associated with TT false positivity, which may arise from non-specific antibody responses or shared epitopes between microbial antigens and treponemal components [19], [23]. A recent large-scale retrospective study by Wang et al. identified several independent risk factors for TTs false positivity, including age years, male gender, and certain clinical specialties such as pediatrics, internal medicine, and nephrology, often associated with immune dysregulation. Additionally, abnormalities in coagulation parameters, such as elevated fibrinogen degradation products and D-dimer, were significantly correlated with false-positive results, suggesting that fibrin degradation products may contribute to assay interference [24]. The case we present here shares the common underlying theme of immune activation with the conditions mentioned above. However, it is distinct in several key aspects. Firstly, unlike the autoantibody-driven interference often seen in SLE, the false positivity in our case was directly attributable to heterophile antibodies, as confirmed by the blocking reagent experiment. Secondly, while other infections can cause false positives, the concurrent false positivity across two different treponemal assays (CLIA and TPPA) specifically due to EBV-induced heterophile antibodies is a phenomenon scarcely reported in the literature.

Furtherly, a critical discrepancy was observed in this case: only Platform A (double-antigen sandwich CLIA, AutoLumo A6000) yielded reactive results, while Platforms B (indirect CLIA, iFlash 3000), C (CMIA, Architect i2000SR), and D (ECLIA, Cobas e801) were consistently non-reactive. Although all four assays are chemiluminescence-based, they differ in assay format, antigen source, and interference-blocking strategies. The dual-antigen sandwich design of Platform A may be particularly susceptible to heterophile antibody interference, as non-specific antibodies can bridge capture and detection antigens, mimicking true treponemal antibody binding. By contrast, indirect CLIA, CMIA, and ECLIA platforms rely on different antigen–antibody–conjugate configurations and include proprietary blocking reagents that may mitigate such effects. These findings highlight that assay-specific variability is clinically relevant and that discordant syphilis serology results across platforms should prompt confirmatory testing to avoid misdiagnosis. While TPPA is often considered more specific and resistant to interference, its performance can still be compromised by excessive antibody cross-reactivity. In this case, heterophile antibodies non-specifically bind to both the *T. pallidum* particle antigens in the TPPA reagent and the antigens in the Platform A dual-antigen sandwich chemiluminescence immunoassay, thereby interfering with the agglutination reaction and chemiluminescence testing.

To our knowledge, this is one of the few reports in which false-positive results on both treponemal-specific assays (TP-Ab and TPPA) were observed in the setting of EBV infection/reactivation and could be reversed after the addition of blocking reagents. The presence of heterophile antibodies, which are known to interfere with serological assays, likely mimicked anti-*Treponema* responses in both treponemal agglutination assays and CLIAs, thereby creating a significant diagnostic pitfall. It illustrates the necessity for multi-platform verification, heterophile blocking confirmation, and careful interpretation of syphilis serology in the broader context of possible viral co-infections.

## Conclusion

This report presents a rare case of false-positive treponemal tests observed in the context of EBV-related infection. It highlights that both treponemal and non-treponemal assays can be affected by non-specific antibody interference, potentially complicating the diagnosis of syphilis. The reversal of this interference upon incubation with a heterophile antibody–blocking reagent not only confirms heterophile antibodies as the definitive cause, but also provides a proof of principle for using such reagents as a confirmatory method in resolving similar diagnostic dilemmas. When treponemal test results are discordant with each other or contradict the clinical probability of syphilis, confirmation with alternative assays and consideration of viral infections like EBV are essential to avoid diagnostic errors and ensure appropriate patient management. However, as a single case report, the generalizability of these findings is limited, and future studies are warranted to elucidate the prevalence and mechanisms of EBV-related serological interference.

**Ethics approval and consent to participate**

.

## Patient consent

Written informed consent was obtained from the patient for publication of this case report and accompanying images. A copy of the written consent is available for review by the Editor-in-Chief of this journal on request.

## Funding

Not applicable.

## Consent for publication

Not applicable.

## CRediT authorship contribution statement

**Chunlei Zhang:** Supervision, Project administration, Data curation. **Qiling Lin:** Methodology, Investigation. **Yi Wu:** Investigation, Data curation. **Mengjie Luo:** Writing – review & editing, Writing – original draft, Methodology, Conceptualization.

## Patient Perspective

The patient expressed initial concern and anxiety over the conflicting test results, fearing a diagnosis of a sexually transmitted infection. Upon receiving a final explanation and reassurance of the EBV-related findings, she was relieved and grateful for the thorough investigation.

## Author Agreement

All authors have participated significantly in the research and writing, and have reviewed and approved the final manuscript.

## Declaration of Competing Interest

The authors declare that they have no conflicts of interest related to the publication of this manuscript. No financial or personal relationships that could influence or bias the work have been identified.

## Data Availability

All data generated or analyzed during this study are included in this article.
