# Supplementary material for: False-positive treponemal syphilis serology linked to EBV-related heterophile antibodies: Insights from a multi-platform diagnostic
Source: IDCases. 2025 Oct 15;42:e02403. doi: 10.1016/j.idcr.2025.e02403 (PMC12593205; doi:10.1016/j.idcr.2025.e02403)
Supplement: Supplementary file 1 — Supplementary material [file mmc1.docx]

**Supplementary materials for the main manuscript**

**Supplementary Table S1. Autoimmune and thyroid antibody profiles**

| Test Item | Result | Unit | Reference Interval | Methodology |
| --- | --- | --- | --- | --- |
| anti-nRNp/Sm | Non-reactive |  | Non-reactive | Western Blot |
| anti-nRNp/Sm | Non-reactive |  | Non-reactive | Western Blot |
| anti-SS-A | Non-reactive |  | Non-reactive | Western Blot |
| Ro-52 | Non-reactive |  | Non-reactive | Western Blot |
| anti-SS-B | Non-reactive |  | Non-reactive | Western Blot |
| anti-Scl-70 | Non-reactive |  | Non-reactive | Western Blot |
| anti-PM-1 | Non-reactive |  | Non-reactive | Western Blot |
| anti-Jo-1 | Non-reactive |  | Non-reactive | Western Blot |
| CENP-B | Non-reactive |  | Non-reactive | Western Blot |
| AnuA | Non-reactive |  | Non-reactive | Western Blot |
| AHA | Non-reactive |  | Non-reactive | Western Blot |
| anti-rRNP | Non-reactive |  | Non-reactive | Western Blot |
| anti-dsDNA | Non-reactive |  | Non-reactive | Western Blot |
| ANA | Non-reactive |  | Non-reactive | Western Blot |
| AMA-M2 | Non-reactive |  | Non-reactive | Western Blot |
| ASO | 50 | IU/mL | < 200 | Immunoturbidimetric assay |
| RF | < 10 | IU/mL | ≤ 30 | Immunoturbidimetric assay |
| CRP | < 0.2 | Mg/L | ≤ 5.00 | Immunoturbidimetric assay |
| TRAb | < 0.800 | IU/mL | ≤ 1.75 | Electrochemiluminescence |
| TPOAb | 12.80 | IU/mL | ≤ 34.00 | Electrochemiluminescence |
| TGAb | 13.80 | IU/mL | ≤ 115.00 | Electrochemiluminescence |
| TG | 29.90 | μg/L | 3.50–77.00 | Electrochemiluminescence |

1. **Laboratory Methods**

Platform A: Treponemal antibody testing was performed on the AutoLumo A6000 automated chemiluminescence immunoassay analyzer (Autobio Diagnostics, China), using a magnetic particle chemiluminescence immunoassay (double-antigen sandwich method). Results were interpreted as reactive when the signal-to-cutoff (S/CO) ratio was ≥1.0 (reference range: S/CO <1.0).

Platform B: Conducted on the iFlash 3000 automated chemiluminescence immunoassay analyzer (Shenzhen YHLO Biotech, China), applying a magnetic particle chemiluminescence immunoassay (indirect method). Reactive results were defined as S/CO ≥1.0 (reference range: S/CO <1.0).

Platform C: Testing was carried out on the Architect i2000SR analyzer (Abbott Laboratories, USA) using a chemiluminescent microparticle immunoassay (CMIA). Results were considered positive when the S/CO ratio was ≥1.0 (reference range: S/CO <1.0).

Platform D: Performed on the Cobas e801 analyzer (Roche Diagnostics, Germany) using an electrochemiluminescence immunoassay (ECLIA). Results were interpreted as reactive when the cutoff index was ≥1.0 (reference range: S/CO <1.0).

*Treponema* pallidum particle agglutination assay (TPPA): Conducted with the TPPA kit (Zhuhai Lizhu Reagent Co., Ltd., China; 100 tests/kit). Agglutination was read visually, with reactive results defined by typical lattice formation according to the manufacturer’s instructions.

Non-treponemal test (TRUST): The toluidine red unheated serum test (TRUST; Rongsheng Biotech, China) was performed according to the manufacturer’s instructions. Reactive results were graded by standard agglutination titers.

Confirmatory and supplementary assays:

Indirect immunofluorescence assay (IFA): Performed using the Anti-*Treponema pallidum* IFA kit (Euroimmun, Germany). Slides were evaluated under a fluorescence microscope, with positive reactivity defined by characteristic fluorescence of *T. pallidum*.

Western blot (WB): Conducted using the Euroline Syphilis Western Blot kit (Euroimmun, Germany). Interpretation followed manufacturer criteria, requiring reactivity against specific *T. pallidum* antigen bands.

Colloidal gold rapid test: The Syphilis Rapid Test Cassette (colloidal gold method; Wantai Biotech, Beijing, China) was employed, with reactive results defined by the appearance of both test and control lines within 15 minutes.

Heterophile antibody blocking assay: To evaluate possible interference, serum was treated with the Heterophilic Blocking Reagent (Scantibodies Laboratory, USA) before repeat testing, following the manufacturer’s protocol.
